# Association between Obesity Indexes and Thyroid Cancer Risk in Korean Women: Nested Case–Control Study

**DOI:** 10.3390/cancers14194712

**Published:** 2022-09-27

**Authors:** Yoonyoung Jang, Taehwa Kim, Brian H. S. Kim, Boyoung Park

**Affiliations:** 1 Department of Preventive Medicine, Hanyang University College of Medicine, Seoul 04763, Korea; 2 Program in Regional Information, Department of Agricultural Economics and Rural Development, Seoul National University, Seoul 08826, Korea; 3 Department of Psychology, Sungkyunkwan University, Seoul 03063, Korea; 4 Program in Agricultural and Forest Meteorology, Research Institute of Agriculture and Life Sciences, Seoul National University, Seoul 08826, Korea

**Keywords:** thyroid cancer in women, nested case–control study, risk factor, obesity

## Abstract

**Simple Summary:**

Regarding the association between obesity and thyroid cancer, most previous studies have focused only on body mass index (BMI), but other measures of obesity have not been studied much with inconsistent results. This study showed a significant association between increased abdominal obesity indexes, such as waist circumference (WC, ≥85.0 cm) and waist-height ratio (WHTR ≥ 0.5) as well as BMI (≥25.0 Kg/m^2^), and increased risk of thyroid cancer. In addition, people who had both abnormally obese levels of BMI and other obesity indexes including WC, waist–hip ratio, or WHTR showed an increased risk of thyroid cancer, compared to those with normal levels of BMI and each obesity index. These results provide evidence of the contribution of both total and central adiposity across the lifespan to thyroid cancer incidence. Risk factor modifications must be considered to explain the current thyroid cancer epidemic.

**Abstract:**

Objective: This study aimed to identify the association between various obesity indexes, including waist circumference (WC), waist–hip ratio (WHR), waist–height ratio (WHTR), and BMI, and their combinations with body mass index (BMI) and thyroid cancer risk. Methods: Of the 65,639 participants who completed a follow-up survey of the Health Examinee Study (HEXA), a prospective cohort of the Korean Genome and Epidemiology Study, 412 female incident thyroid cancer cases, and 1648 birth year- and enrollment year-matched female controls were included. Multiple conditional logistic regression was used to estimate the association between obesity indexes and thyroid cancer risk. Results: The risk of developing thyroid cancer was increased by 1.37-fold (95% confidence interval (CI) = 1.03–1.81) higher in the obese BMI group (≥25.0 Kg/m^2^) compared to that in the normal BMI group (<23.0 Kg/m^2^). Obesity in terms of WC (≥85.0 cm) and WHTR (≥0.5) was associated with an increased risk of thyroid cancer (OR 1.55, 95% CI = 1.16–2.07; OR 1.37, 95% CI = 1.07–1.75, respectively). However, increased WHR levels did not show any significant association. Women with both obese levels of BMI (≥25.0 Kg/m^2^) and other obesity indexes (WC ≥ 85.0 cm, WHR ≥ 0.85, or WHTR ≥ 0.5) showed an increased risk of thyroid cancer with OR of 1.63 (95% CI = 1.14–2.31), 1.49 (95% CI = 1.05–2.12), and 1.42 (95% CI = 1.04–1.94), compared to those with normal levels of BMI and each obesity index. Conclusion: These results provide evidence of the contribution of both total and central adiposity across the lifespan of thyroid cancer incidence. Risk factor modifications must be considered to explain the current thyroid cancer epidemic.

## 1. Introduction

In 2020, 19.3 million incident cases of cancer and 10 million cancer-related deaths were estimated worldwide. Among the cancer types, 586,000 thyroid cancers were developed, which were ranked ninth. One of the unique characteristics of thyroid cancer is the higher incidence in women than in men, with age-standardized incidence rates (ASR) in women of 10.1 per 100,000 vs. that in men of 3.1 per 100,000 worldwide. The incidence of thyroid cancer is higher in developed countries than in developing countries. In particular, females in developed countries have approximately 5.5 times higher incidence rates of thyroid cancer than that of women in developing countries [1].

Thyroid cancer is the most common cancer in Korea. Despite the rapid decrement between 2012 and 2015 after the issue of overdiagnosis emerged, the incidence of thyroid cancer has shown an increasing trend again since 2015. The pattern of thyroid cancer incidence in Korea has mainly been due to the incidence pattern of women. A relative survival of more than 100%, a high correlation between thyroid cancer incidence and screening rate, and a prominent period effect in Korea have raised overdiagnosis issues as a major cause of the higher incidence of thyroid cancer [2,3].

Despite a growing understanding of the impact of overdiagnosis on thyroid cancer, the etiology of thyroid cancer is not well understood. In addition to ionizing radiation exposure in children as a well-known risk factor, iodine intake, obesity, diabetes, estrogen, reproductive factors, autoimmune thyroiditis, and several lifestyle factors have been suggested as risk factors for thyroid cancer. However, these suggested risk factors have not drawn conclusive results [4].

Among the Korean population where the thyroid cancer epidemic has been identified, studies regarding risk factors of thyroid cancer suggested family history, obesity, non-smoking, non-drinking, and low income as risk factors for thyroid cancer [5,6,7]. Among these results, the association between obesity and thyroid cancer risk has been the most studied. However, most of these were case–control studies and focused only on body mass index (BMI), which cannot distinguish the proportion of weight between fat and muscle; thus, the role of body fat in thyroid cancer risk could not be confirmed [8,9,10].

Therefore, this study investigated the association of various obesity indexes, including waist circumference (WC), waist–hip ratio (WHR), and waist–height ratio (WHTR), as well as BMI and their combinations, with BMI and thyroid cancer in the Korean population, based on a prospective study design in Korean women.

## 2. Material and Methods

### 2.1. Study Population

We analyzed the baseline and follow-up data from the Health Examinee Study (HEXA), a prospective cohort of the Korean Genome and Epidemiology Study (KoGES). The KoGES has been established to investigate genetic and environmental factors and their interactions in the development of chronic and complex diseases in Korea [11]. The study population of HEXA was composed of people aged 40–69 years who were recruited between 2004 and 2014 at 38 large health examination centers and training hospitals in eight regions of Korea. A total of 173,353 patients were recruited, and 65,639 completed the follow-up studies between 2012 and 2017 [12].

Cancer cases were ascertained based on a self-reported diagnosis of cancer by a physician through a standardized questionnaire administered by trained interviewers. Of the 65,639 participants who completed the follow-up study, thyroid cancer development was identified through a follow-up questionnaire. To exclude prevalent cancer cases, subjects who had been diagnosed with any type of cancer by physicians at the baseline survey (*n* = 6038) were excluded. Participants with missing information on variables used to calculate obesity indices (height, weight, waist circumference, or hip circumference) were excluded (*n* = 313). In addition, in the follow-up survey, people who reported developing cancers other than thyroid cancer (*n* = 1155) were excluded, with 456 thyroid cancer development cases and 61,187 cancer-free participants finally remaining.

The positive predictive value of self-reported thyroid cancer in the HEXA baseline study was 96.1% [13]. Another prospective cohort study in Korea showed that the accuracy of self-reported incident thyroid cancer was 98.1% and 99.8% in terms of sensitivity and specificity, respectively [5]. Thus, the self-reported information regarding the diagnosis of thyroid cancer by physicians is valid. HEXA provided informed consent for the baseline and follow-up data from all participants and then conducted interviews and physical examinations [12]. This study was approved by the Institutional Review Board of Hanyang University College of Medicine (IRB No. HYUIRB-202205-025).

### 2.2. Nested Case–Control Data Set

**Figure 1:**
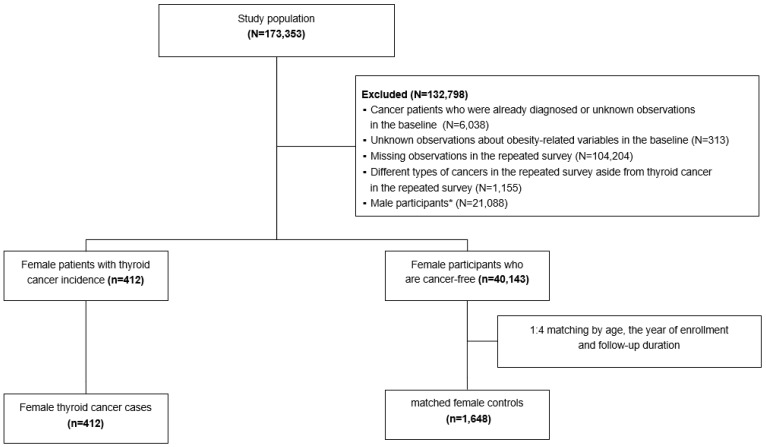
Flow chart of selection of the study participants. * Excluded male participants because the number of male thyroid cancer cases was too small.

A total of 456 thyroid cancer cases were identified, including 412 women and 44 men. Controls were randomly selected from participants who were free from any type of cancer in both the baseline and follow-up questionnaires. Four controls were matched to each case according to the year of birth (exactly matched) and the enrollment year (± 1 year). The follow-up duration was calculated by subtracting the enrollment year from the follow-up year. The follow-up duration of the matched controls was set to be the same or longer than that of each thyroid cancer case. Because the number of male thyroid cancer cases was too small, 412 female thyroid cancer cases and matched 1648 female controls were included in the final analysis (Figure 1). 

### 2.3. Definition of Variables

During the health examination, anthropometric indices, including height, weight, waist circumference, and hip circumference, were measured by trained nurses [12]. Information on obesity indexes pertained to BMI, WC, WHR, and WHTR. BMI was classified as obese (≥25.0 Kg/m^2^), overweight (23.0–24.9 Kg/m^2^), and normal (<23.0 Kg/m^2^). Participants with WC ≥ 85 cm, WHR ≥ 0.85, and WHTR ≥ 0.5 were considered obese, and participants with WC < 85, WHT < 0.85, and WHTR < 0.5 were considered normal [14,15].

Through a standardized questionnaire administered by trained interviewers, participants were asked to provide information on the diagnosis of comorbidities by physicians [12], the medical history of their first-degree relatives, lifestyle-related factors, and reproductive factors. Lifestyle-related factors, including drinking (never, former, or current), smoking (never, former, or current), sweating exercise more than once a week (no, yes), reproductive factors including hysterectomy (yes or no), oral contraceptive use (never, former, or current), and subjective health evaluation (good, normal, or bad), were considered covariates. Of the comorbidities, the diagnosis of hypertension, diabetes, hyperlipidemia, osteoporosis, and intestinal polyps, which are associated with both obesity and thyroid cancer, were considered covariates [16,17,18,19,20,21]. In addition, a family history of cancer, hypertension, and diabetes in first-degree relatives were considered covariates [22]. Fasting blood glucose measured from blood samples during the course of health examination was classified into <100 and ≥100 mg/dl [23].

### 2.4. Statistical Analysis

Characteristics that were measured at the baseline survey between the thyroid cancer cases and matched control groups were presented as proportions or means and compared using a chi-square or Fisher’s exact test for categorical variables and a *t*-test for continuous variables. A conditional logistic regression model was applied to determine the association between BMI, WC, WHR, WHTR, and thyroid cancer risk. In addition, multiple conditional logistic regression models were applied and adjusted for drinking, smoking, sweating exercise, past medical history of hypertension, diabetes, hyperlipidemia, osteoporosis, polyp of the intestine, hysterectomy, oral contraceptive use, family history of cancer, hypertension, diabetes, fasting blood sugar, and subjective health evaluation. The association of WC, WHR, WHTR, and BMI with thyroid cancer was analyzed by conditional logistic regression, adjusted for the above covariates with reference to normal obesity indexes in both BMI and WC, WHR, and WHTR. Statistical significance was defined as a two-sided *p*-value <0.05. Statistical analyses were performed using the SAS statistical software (version 9.4; SAS Institute, Cary, NC, USA).

## 3. Results

The baseline characteristics of incident thyroid cancer cases and matched controls are shown in Table 1. The proportion of patients with a history of osteoporosis, intestinal polyps, and hysterectomy was higher among patients with thyroid cancer than in the matched controls (9.0% vs. 5.8%, 4.8% vs. 2.4%, and 15.5% vs. 10.2%, *p*-value < 0.05). The mean fasting blood sugar was higher in thyroid cancer cases compared to that in matched controls (94.0 vs. 91.0 mg/dl, *p*-value = 0.0039). Incident thyroid cancer patients reported worse subjective health statuses at baseline (22.1% vs. 16.4%). Other characteristics in incident thyroid cancer cases and matched controls were not significantly different. 

**Figure 2:**
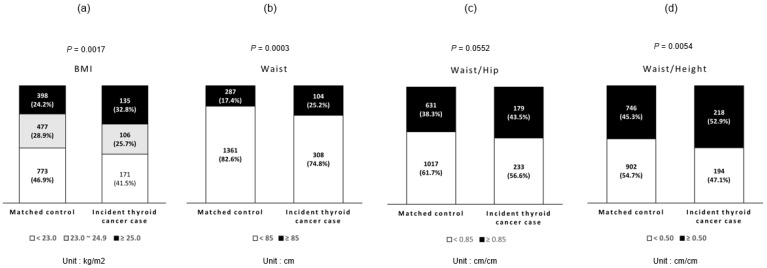
Prevalence of obesity in baseline measurements. (**a**) The proportions of obesity in terms of BMI. (**b**) The proportions of obesity in terms of WC. (**c**) The proportions of obesity in terms of WHR. (**d**) The proportions of obesity in terms of WHTR. BMI was classified into obese (≥25.0 Kg/m^2^), overweight (23.0–24.9 Kg/m^2^), and normal (<23.0 Kg/m^2^). Participants with WC ≥ 85 cm, WHR ≥ 0.85, and WHTR ≥ 0.5 were considered obese, and participants with WC < 85, WHT < 0.85, and WHTR < 0.5 were considered normal. *p*-Values were estimated from chi-square test. The proportions of obesity in terms of BMI, WC, and WHTR in thyroid cancer cases were significantly higher than matched controls.

The proportions of obesity in terms of BMI, WC, WHR, and WHTR are shown in Figure 2. The proportions of obese and overweight patients with BMI were 32.8% and 25.7% in thyroid cancer cases and 24.2% and 28.9% in matched controls, respectively (*p*-value = 0.0017). The proportions of obesity in terms of WC, WHR, and WHTR in thyroid cancer cases were 25.2%, 43.5%, and 52.9%, respectively, and those of matched controls were 17.4%, 38.3%, and 45.3%, respectively, with a significantly higher proportion of WC and WHTR in thyroid cancer cases.

The associations between the obesity indexes and thyroid cancer risk are shown in Table 2. Compared with that of the normal BMI group (<23.0 Kg/m^2^), the risk of developing thyroid cancer was increased 1.37-fold (95% CI = 1.03–1.81) in the obese BMI group (≥25.0 Kg/m^2^) after adjusting for other covariates. Obesity in terms of WC (≥85.0 cm) and WHTR (≥0.5) was associated with an increased risk of thyroid cancer (OR 1.55, 95% CI = 1.16–2.07; OR 1.37, 95% CI = 1.07–1.75, respectively). Abdominal obesity in the WHR group (≥0.85) was not significantly associated with thyroid cancer risk. 

Table 3 shows the association between the combination of BMI, other obesity indexes, and thyroid cancer risk. Women with both obese levels of BMI (≥25.0 Kg/m^2^) and other obesity indexes (WC ≥ 85.0 cm, WHR ≥ 0.85 or WHTR ≥ 0.5) showed an increased risk of thyroid cancer with ORs of 1.63 (95% CI = 1.14–2.31), 1.49 (95% CI = 1.05–2.12), and 1.42 (95% CI = 1.04–1.94), compared to those with a normal level of BMI and each obesity index. However, women with increased levels of only one obesity index did not have a significantly increased risk of thyroid cancer. 

## 4. Discussion

In this study, obesity indexes including BMI, WC, and WHTR were associated with an increased risk of thyroid cancer in Korean women. When obesity indexes including WC, WHR, and WHTR were combined with the BMI category, women with both obesity-level BMI and other obesity indexes showed a significantly increased risk of thyroid cancer compared to that of women with normal BMI and other obesity indexes. 

Previous studies have shown that obesity and overweight are associated with an increased risk of thyroid cancer [8,9,10]. Although there were differences in the categories of obesity, strength of the association, and some heterogeneity of the dose–response pattern between men and women, the International Agency for Research on Cancer concluded that there is sufficient evidence that body fatness, defined as BMI category, increases thyroid cancer risk by 1.1-fold per 5 BMI units [24]. In an Asian cohort consortium study, the association between increased BMI and thyroid cancer risk was stronger in men than in women [25]. This study did not include men due to the small number of thyroid cancer cases in men, but the association between BMI and thyroid cancer risk in women was comparable to that reported in previous studies. Epidemiological research collecting body measurements, apart from height and weight, rarely exists. BMI is a common and useful obesity-related index but cannot show lean body mass or central and peripheral body fat distribution [26]. 

The associations between other obesity indexes, except BMI and thyroid cancer risk, were studied for WC, WHR, or WHTR; however, the number of studies was limited, and the results were inconsistent [26,27,28,29]. The European Prospective Investigation into Cancer and Nutrition study of 10 countries identified that increased WC and WHR were significantly associated with thyroid cancer risk in females, but not in males. Height and leg length were related to thyroid cancer in males [29]. In contrast, a study of postmenopausal women in the United States showed that WC and WHR were not significantly associated with thyroid cancer. Otherwise, a taller height is related to the risk of thyroid cancer [27]. Another study using the NIH-AARP Diet and Health Study showed that large WC was significantly associated with increased thyroid cancer risk in both men and women [28]. A recent pooled analysis of 22 prospective studies identified that not only BMI but also WC and height were associated with an increased risk of thyroid cancer with an HR of 1.03 per 5 cm increment of WC and 1.07 per 5 cm increment of height [26]. In Asia, despite the lack of studies on obesity indexes other than BMI and thyroid cancer risk, studies related to thyroid nodules showed that WC was positively associated with the prevalence of thyroid nodules [30]. Of the available obesity indices, BMI, which provides information about total body fat, has been most frequently used to determine the obesity status in general populations. However, more recently, indices that reflect central obesity, such as WC, WHR, and WHTR, have been suggested to provide a more accurate indication of abdominal body fat when compared with BMI. Additionally, central obesity indices are said to be associated with disease risk to a greater extent than BMI [31,32,33,34]. This study showed a significant association between increased abdominal obesity indexes, such as WC or WHTR, and an increased risk of thyroid cancer in East Asian women with a thyroid cancer epidemic having emerged. 

Only a few studies have assessed the association between obesity indices and the risk of thyroid cancer. A study showed that people who had both abnormally obese levels of BMI and WC were much more vulnerable to thyroid cancer than that of the reference group with normal BMI and WC [28]. We found that only combined variables with both obese level BMI and abdominal obesity (WC, WHR, and WHTR) were statistically significant for the incidence of thyroid cancer. If only one of the indexes was obese with either the BMI or abdominal obesity index, these combinations were not significant. These results could add evidence to the contribution of both total and central adiposity across the lifespan to thyroid cancer incidence. 

The biological mechanisms underlying thyroid cancer and obesity have not yet been clearly identified. Resistance to insulin, altered adipocytokine components, inflammatory response, and their combinations have been suggested as mechanisms underlying the relationship between obesity and thyroid cancer risk. Endocrine disorders and inflammation of central obesity-related adipose tissue were observed in obese individuals, leading to insulin resistance. High thyrotropin hormone levels have been identified in overweight and obese individuals, and thyrotropin is associated with mitogenic effects. In addition, high levels of serum tumor necrosis factor-alpha (TNF-α), interleukin-6, TNF-α immunoreactivity, and leptin hormone in thyroid tissues have been demonstrated in patients with thyroid cancer [25].

The main limitation of this study was that the proportion of participants in both the baseline and follow-up surveys was low. Therefore, selection bias due to loss of follow-up may have affected the results. However, when we compared the baseline characteristics between women who did or did not participate in the follow-up survey, no differences were identified. Thus, we expected that the effect of selection bias would be minimal. Second, newly incident thyroid cancer was assessed using a questionnaire at the follow-up [12]. A previous study showed that self-reported cancer history in the HEXA study had a high accuracy [13], and another study in Korea also showed a high validity of self-reported cancer information [5]. Other comorbidities that were applied were measured through questionnaires, for which the validity of the collected information may be biased. However, considering the high accuracy of medical histories obtained via self-reported questionnaires [35], the information regarding comorbidities in this study, which was measured by trained interviewers, would be accurate. Third, this study could not consider the subtypes or other markers of thyroid cancer because of the unavailability of information. However, a previous pooled analysis showed that excess body fat was associated with an increased risk of most major types of thyroid cancer [26]. Possible confounding factors or major risk factors for thyroid cancer, such as ionizing radiation exposure or iodine intake, were not measured and could not be adjusted. In addition, information about the covariates was obtained based on self-reports in the survey, and the accuracy of the information was limited. Despite these limitations, to the best of our knowledge, this study is the first to investigate the association between a combination of obesity indexes and thyroid cancer risk, suggesting that a combination of general obesity in terms of BMI and abdominal obesity could elevate risk. 

## 5. Conclusions

Despite overdiagnosis, thyroid cancer is the most common cancer in Korea, with a high disease burden, and increased thyroid cancer incidence has been observed in many countries. Thus, risk factor modifications should be considered to explain the current thyroid cancer epidemic. The concurrent increase in obesity prevalence and thyroid cancer incidence and evidence from epidemiological studies raise the question of the contribution of obesity or other risk factors to thyroid cancer carcinogenesis. Further studies should identify the possible causes of the thyroid cancer epidemic in terms of risk factors.

## Figures and Tables

**Table 1:** Baseline characteristics of incident thyroid cancer patients and matched controls.

Characteristic	Thyroid Cancer Cases ^1^ (*n* = 418)	Matched Controls ^1^ (*n* = 1648)	*p*-Value
Age at cohort enrollment			
Mean (standard deviation)	51.0 (±6.7)	51.0 (±6.7)	1.000
40–49	183 (44.4)	732 (44.4)	1.000
50–59	175 (42.5)	700 (42.5)	
≥60	54 (13.1)	216 (13.1)	
Alcohol drinking			
Never	272 (66.0)	1077 (65.4)	0.3970
Former or current	135 (32.8)	561 (34.0)	
Unknown	5 (1.2)	10 (0.6)	
Smoking			
Never	397 (96.4)	1596 (96.8)	0.1358
Former or current	9 (2.2)	43 (2.6)	
Unknown	6 (1.4)	9 (0.6)	
Sweating exercise once or more per week			
No	170 (41.3)	750 (45.5)	0.2110
Yes	242 (58.7)	896 (54.4)	
Unknown	0 (0.0)	2 (0.1)	
Past medical history of hypertension			
No	348 (84.5)	1420 (86.2)	0.2616
Yes	63 (15.3)	227 (13.7)	
Unknown	1 (0.2)	1 (0.1)	
Past medical history of diabetes			
No	394 (95.6)	1586 (96.3)	0.6626
Yes	18 (4.4)	60 (3.6)	
Unknown	0 (0.0)	2 (0.1)	
Past medical history of hyperlipidemia			
No	379 (92.0)	1508 (91.5)	0.7507
Yes	33 (8.0)	140 (8.5)	
Past medical history of osteoporosis			
No	375 (91.0)	1553 (94.2)	0.0171
Yes	37 (9.0)	95 (5.8)	
Past medical history of polyp of intestine			
No	392 (95.2)	1608 (97.6)	0.0088
Yes	20 (4.8)	40 (2.4)	
Hysterectomy			
No	346 (84.0)	1474 (89.4)	0.0083
Yes	64 (15.5)	168 (10.2)	
Unknown	2 (0.5)	6 (0.4)	
Use oral contraceptive			
Never	324 (78.6)	1340 (81.3)	0.2267
Former or current	82 (19.9)	296 (18.0)	
Unknown	6 (1.5)	12 (0.7)	
Family history of cancer			
No	267 (64.8)	1150 (69.8)	0.1208
Yes	143 (34.7)	487 (29.6)	
Unknown	2 (0.5)	11 (0.6)	
Family history of hypertension			
No	266 (64.6)	1105 (67.1)	0.5224
Yes	145 (35.2)	536 (32.5)	
Unknown	1 (0.2)	7 (0.4)	
Family history of diabetes			
No	313 (76.0)	1312 (79.6)	0.1560
Yes	97 (23.5)	322 (19.5)	
Unknown	2 (0.5)	14 (0.9)	
Fasting blood sugar			
Mean (standard deviation)	94.0 (±20.6)	91.0 (±16.5)	0.0039
<100 mg/dl	327 (79.4)	1370 (83.1)	0.0861
≥100 mg/dl	74 (18.0)	226 (13.7)	
Unknown	11 (2.6)	52 (3.2)	
Subjective health evaluation			
Good	127 (30.8)	578 (35.1)	0.0463
Normal	192 (46.6)	788 (47.8)	
Bad	91 (22.1)	271 (16.4)	
Unknown	2 (0.5)	11 (0.7)	

^1^ Thyroid cancer cases and controls were matched by sex (exactly matched), year of birth (exactly matched), and enrollment year (±1 year). The follow-up duration of the matched controls was set to be the same or longer than that of each thyroid cancer case.

**Table 2:** Association between obesity indices and thyroid cancer risk.

Obesity Index	Crude Odds Ratio	95% CI	*p*-Value	Adjusted Odds Ratio ^†^	95% CI	*p*-Value
BMI						
≥25.0 Kg/m^2^	1.46	1.12–1.91	0.0052	1.37	1.03–1.81	0.0300
23.0–24.9 Kg/m^2^	0.96	0.73–1.27	0.7896	0.96	0.72–1.28	0.8000
<23.0 Kg/m^2^	1			1		
Waist circumference						
≥85 cm	1.65	1.25–2.17	0.0004	1.55	1.16–2.07	0.0035
<85 cm	1			1		
Waist–hip ratio						
≥0.85	1.25	0.98–1.58	0.0706	1.20	0.93–1.54	0.1529
<0.85	1			1		
Waist–height ratio						
≥0.50	1.41	1.11–1.78	0.0045	1.37	1.07–1.75	0.0135
<0.50	1			1		

CI, confidence interval; ^†^ Adjusted for hypertension (yes, no), diabetes (yes, no), hyperlipidemia (yes, no), osteoporosis (yes, no), polyp of the intestine (yes, no), familial cancer (yes, no), familial hypertension (yes, no), familial diabetes (yes, no), drinking (never, former, or current), smoking (never, former, or current), intense exercise (no, yes), hysterectomy (yes, no), oral contraceptive (never, former, or current), fasting blood sugar (<100, ≥100), and subjective health evaluation (good, normal, bad).

**Table 3:** Associations between abdominal obesity, BMI, and thyroid cancer risk.

Parameter	Crude Odds Ratio	95% CI	*p*-Value	Adjusted Odds Ratio ^†^	95% CI	*p*-Value
BMI & waist circumference						
≥25.0 Kg/m^2^ & ≥85 cm	1.79	1.29–2.49	0.0005	1.63	1.14–2.31	0.0070
23.0–24.9 Kg/m^2^ & ≥85 cm	1.36	0.76–2.42	0.2989	1.39	0.76–2.52	0.2863
<23.0 Kg/m^2^ & ≥85 cm	1.28	0.51–3.23	0.6046	1.36	0.53–3.53	0.5261
≥25.0 Kg/m^2^ & <85 cm	1.20	0.84–1.71	0.3072	1.18	0.82–1.70	0.3719
23.0–24.9 Kg/m^2^ & <85 cm	0.92	0.69–1.24	0.5927	0.93	0.68–1.26	0.6297
<23.0 Kg/m^2^ & <85 cm	1			1		
BMI & waist–hip ratio						
≥25.0 Kg/m^2^ & ≥0.85	1.60	1.15–2.22	0.0055	1.49	1.05–2.12	0.0266
23.0–24.9 Kg/m^2^ & ≥0.85	1.07	0.72–1.59	0.7346	1.05	0.70–1.57	0.8140
<23.0 Kg/m^2^ & ≥0.85	1.21	0.82–1.81	0.3392	1.23	0.82–1.84	0.3229
≥25.0 Kg/m^2^ & <0.85	1.45	0.98–2.16	0.0633	1.37	0.91–2.07	0.1269
23.0–24.9 Kg/m^2^ & <0.85	0.97	0.68–1.39	0.8716	0.99	0.69–1.43	0.9685
<23.0 Kg/m^2^ & <0.85	1			1		
BMI & waist–height ratio						
≥25.0 Kg/m^2^ & ≥0.5	1.51	1.13–2.02	0.0059	1.42	1.04–1.94	0.0267
23.0–24.9 Kg/m^2^ & ≥0.5	1.20	0.85–1.70	0.2893	1.20	0.85–1.72	0.3023
<23.0 Kg/m^2^ & ≥0.5	1.11	0.71–1.74	0.6577	1.10	0.69–1.76	0.6757
≥25.0 Kg/m^2^ & <0.5	1.46	0.74–2.87	0.2712	1.30	0.64–2.65	0.4621
23.0–24.9 Kg/m^2^ & <0.5	0.74	0.50–1.12	0.1524	0.75	0.49–1.13	0.1660
<23.0 Kg/m^2^ & <0.5	1			1		

CI, confidence interval; ^†^ Adjusted for hypertension (yes, no), diabetes (yes, no), hyperlipidemia (yes, no), osteoporosis (yes, no), polyp of the intestine (yes, no), familial cancer (yes, no), familial hypertension (yes, no), familial diabetes (yes, no), drinking (never, former, or current), smoking (never, former, or current), intense exercise (no, yes), hysterectomy (yes, no), oral contraceptive (never, former, or current), fasting blood sugar (<100, ≥100), and subjective health evaluation (good, normal, bad).

## Data Availability

The data in this study are available from The Health Examinees (HEXA) study (https://kdca.go.kr/contents.es?mid=a40504060100, accessed on 1 February 2022) by submitting the study protocol, IRB approval certification, and mandatory information.
